# Temperature and predator-mediated regulation of plasma cortisol and brain gene expression in juvenile brown trout (*Salmo trutta*)

**DOI:** 10.1186/s12983-020-00372-y

**Published:** 2020-08-28

**Authors:** Karl Filipsson, Eva Bergman, Larry Greenberg, Martin Österling, Johan Watz, Ann Erlandsson

**Affiliations:** River Ecology and Management, Department of Environmental and Life Sciences, Karlstad University, Universitetsgatan 2, SE-651 88 Karlstad, Sweden

**Keywords:** Anti-predator, Burbot, Climate change, HPI axis, mRNA, Salmonid, Stress, Winter

## Abstract

**Background:**

Temperature affects many aspects of performance in poikilotherms, including how prey respond when encountering predators. Studies of anti-predator responses in fish mainly have focused on behaviour, whereas physiological responses regulated through the hypothalamic-pituitary-interrenal axis have received little attention. We examined plasma cortisol and mRNA levels of stress-related genes in juvenile brown trout (*Salmo trutta*) at 3 and 8 °C in the presence and absence of a piscivorous fish (burbot, *Lota lota*).

**Results:**

A redundancy analysis revealed that both water temperature and the presence of the predator explained a significant amount of the observed variation in cortisol and mRNA levels (11.4 and 2.8%, respectively). Trout had higher cortisol levels in the presence than in the absence of the predator. Analyses of individual gene expressions revealed that trout had significantly higher mRNA levels for 11 of the 16 examined genes at 3 than at 8 °C, and for one gene (retinol-binding protein 1), mRNA levels were higher in the presence than in the absence of the predator. Moreover, we found interaction effects between temperature and predator presence for two genes that code for serotonin and glucocorticoid receptors.

**Conclusions:**

Our results suggest that piscivorous fish elicit primary stress responses in juvenile salmonids and that some of these responses may be temperature dependent. In addition, this study emphasizes the strong temperature dependence of primary stress responses in poikilotherms, with possible implications for a warming climate.

## Background

Outcomes of predator-prey interactions depend on the relative performance of predator and prey, which can be strongly temperature dependent in poikilotherms [1–4]. Therefore, temperature is predicted to have strong effects on anti-predator responses [1, 3–5]. Prey fishes, such as juvenile salmonids, typically respond to the presence of piscivorous fish [6–12] and the strength of these responses may change with water temperature [13–17]. Temperature effects on anti-predator responses in fish have mainly been studied in tropical and warm-temperate systems and rarely under winter conditions [12]. Although temperatures all over the Earth are increasing as a result of climate change, global warming is pronounced in boreal and Arctic regions, especially during the winter season [18–23]. Hence, elevated winter temperatures can possibly have major effects on anti-predator responses of fishes.

Physiological stress is an adaptive response that allows animals to react to disturbance, such as predation threat [24, 25]. Primary stress responses include neuroendocrine regulation through the hypothalamic-pituitary-interrenal (HPI) axis [24, 26], through which corticotropin releasing factor (CRF) induces the release of cortisol into the circulatory system [26, 27]. Cortisol acts through glucocorticoid receptors (GR) and mineralocorticoid receptors (MR) [24], which are involved in modulation of the stress response and results in behavioural changes [28–30]. Cortisol levels generally increase in fish in the presence of predators [31–35], and fish from areas with high and low predation pressure can exhibit differences in both cortisol regulation and behaviour [36, 37]. The serotonergic (5-HT) system is also associated with the HPI axis. Increased serotonergic activity is involved in stress responses in teleosts [38–40] and has subsequent effects on behaviour [38, 39, 41, 42]. Changes in serotonergic activity have been observed in fish in the presence of predators [43, 44]. Cortisol and serotonin levels in fish can also increase as a response to thermal stress [24, 45–47]. In addition, arginine-vasotocin (AVT) regulation occurs through the HPI axis [48] and is strongly associated with social and aggressive behaviours in teleosts [49–52].

Secondary stress responses include effects on metabolism and immune function [24, 27, 53]. Immune responses are activated when stressors are short-term and acute, whereas chronic stressors result in suppressed immune function [53, 54]. Expressions of genes associated with immune function, such as calmodulin (CALM), gamma-aminobutyric acid receptor-associated protein (GABARAP), major histocompatibility complex I (MHC1) and retinol binding protein (RPB1), may therefore be affected by external stressors. Regulation of ependymin (EPD1), the most abundant glycoprotein in fish cerebrospinal fluid [55], is affected by environmental stress and involved in cold acclimation [56, 57]. CALM, EPD1, GABARAP and RBP1 are also involved in neural and behavioural plasticity [30, 55, 58, 59], and previous work shows that regulation of all the above mentioned genes are involved in stress responses in salmonids [60, 61]. For example, rainbow trout (*Oncorhynchus mykiss*) have higher mRNA levels of EPD1, GABARAP and CRF after a simulated attack from an avian predator [62]. Gene expressions of antioxidative stress proteins such as glutathione reductase (GSR), although not regulated through the HPI axis, may also be affected by environmental stressors, which has been shown in brown trout (*Salmo trutta*) exposed to heavy metals [63].

Whole-animal responses to physiological stress, such as behaviour and growth, are often termed tertiary responses [64, 65]. Secondary and tertiary stress responses in the presence of predators are well studied in salmonids [11, 12, 66–68], whereas primary responses, i.e. gene expression and endocrine regulation, have not received as much attention (but see [31, 62, 69]). To understand the mechanisms underlying anti-predator behaviours in fish, studies of primary physiological stress responses and their associated gene expressions could provide valuable insight into the direct effects of environmental stressors. Hence, studies of genes associated with stress such as CRF, as well as cortisol, 5-HT and AVT receptor genes, may elucidate how fish respond to changes in water temperature and natural stressors such as predation risk.

The purpose of this study was to examine physiological stress responses in a juvenile salmonid at different winter temperatures in the presence and absence of a predator. Specifically, we examined plasma cortisol levels and brain gene expression (measured as relative mRNA levels) for stress-related genes in juvenile brown trout at 3 and at 8 °C in the presence and absence of burbot (*Lota lota*), a winter-active piscivorous fish. We predicted that trout would exhibit increased cortisol levels in the presence of burbot, thus indicating physiological stress. Likewise, we predicted that telencephalic gene expressions would be higher in the presence of burbot than in its absence, and that the expressions might be temperature dependent.

## Results

### Multivariate analysis

**Fig. 1:**
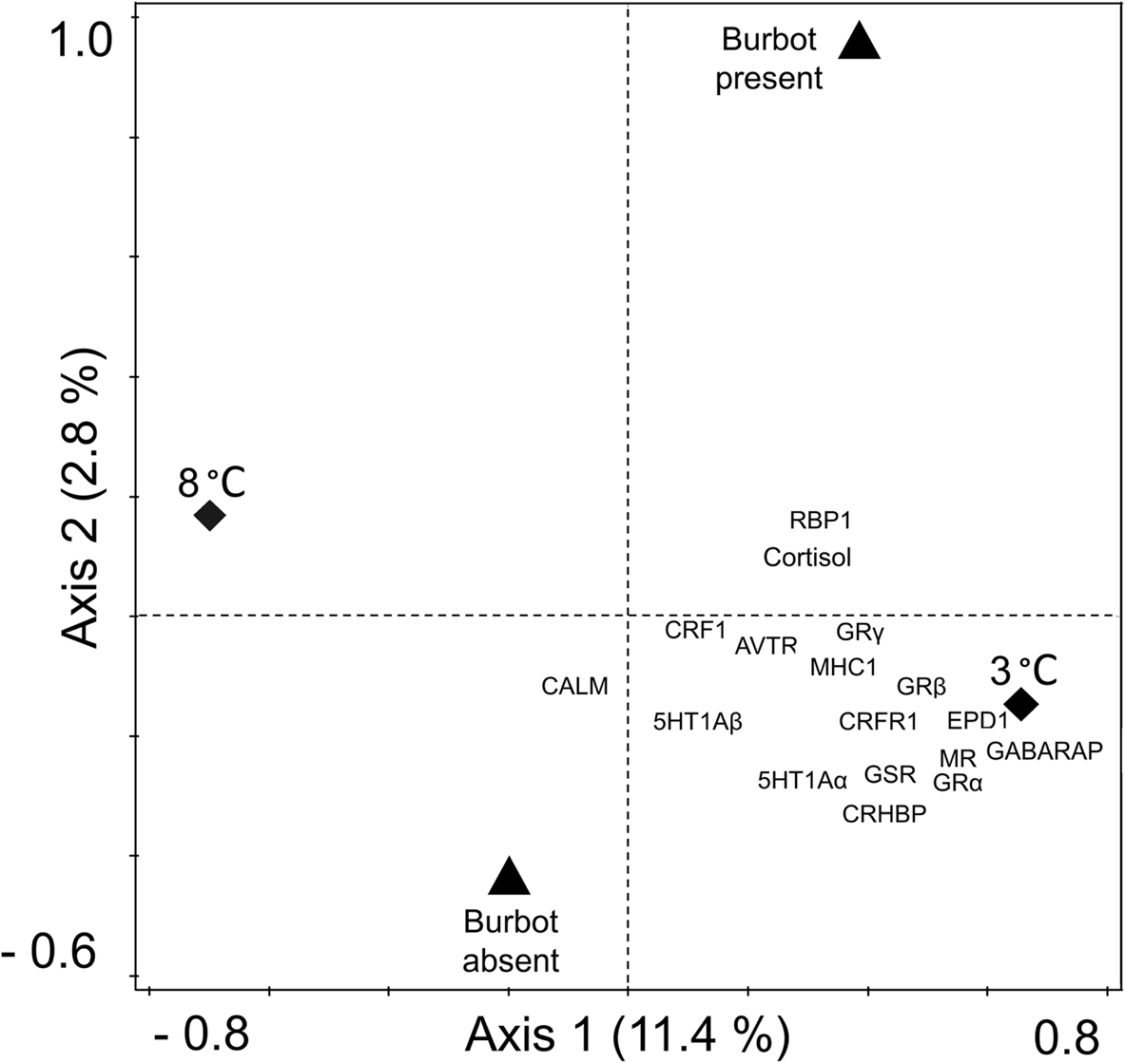
Ordination plot of the redundancy analysis (RDA). The RDA was constructed using gene expression (mRNA levels) and cortisol data from juvenile brown trout (*Salmo trutta*) (*N* = 79, see Table 3) subjected to two experimental treatments (water temperature and presence/absence of predatory burbot (*Lota lota*)). The two temperatures (3 and 8 °C) drive the variation on axis one and explain 11.4% of the total variation in the data. Axis two is driven by burbot presence/absence and explains 2.8% of the total variation. The position of each response variable in relation to the intersection of the two orthogonal axes indicates the direction of the positive correlation, whereas the distance between intersect and each variable describes the strength of the effect. Biplot arrows have been removed for clarity

The first axis of the redundancy analysis (RDA) was driven by the temperature treatment, which explained a significant amount of the observed variation in cortisol and mRNA levels (11.4% of all variation, *F*_*1, 69*_ = 9.7, *P* = 0.002). Predator treatment explained the variation on the second axis. The presence or absence of a burbot explained less variation than the temperature treatment, albeit a significant amount (2.8% of all variation, *F*_*1, 69*_ = 2.8, *P* = 0.03) (Fig. 1).

### Cortisol

**Fig. 2:**
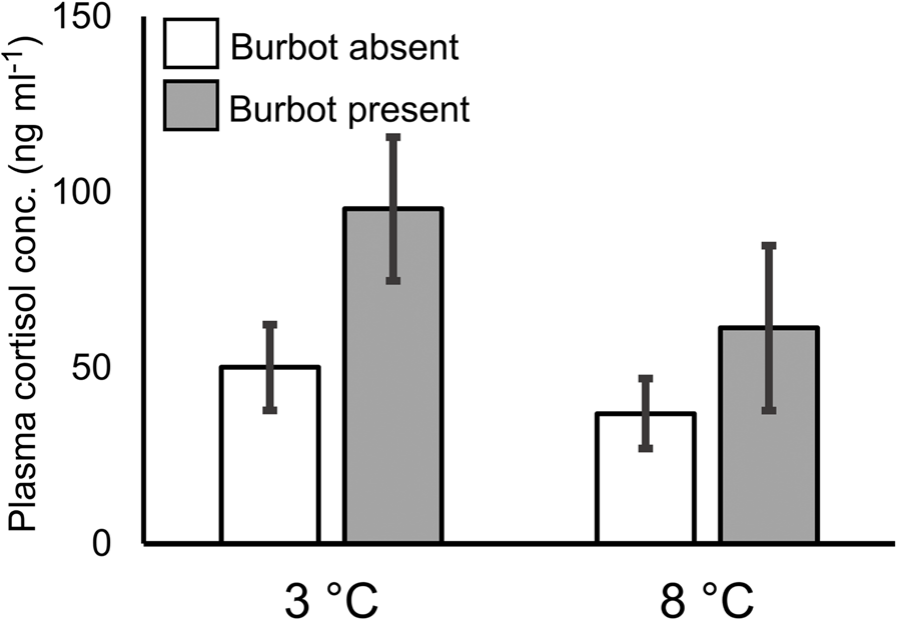
Mean plasma cortisol concentrations in juvenile brown trout (*Salmo trutta*) (*N* = 80, see Table 3) at 3 and 8 °C in the presence and absence of predatory burbot (*Lota lota*). Two-way ANOVA revealed that the presence of burbot had a significant effect on trout plasma cortisol levels (*P* < 0.01), regardless of temperature treatment. Error bars denote ±1 s.e

There was a significant effect of predator presence on trout plasma cortisol levels (Table 1). Trout had higher cortisol levels (mean ± 1 s.e.) in the presence of the predator (82.6 ± 15.9 ng ml^− 1^) than in its absence (45.7 ± 7.6 ng ml^− 1^) (Fig. 2). Temperature and the temperature × predator interaction had no significant effect on cortisol levels (Table 1).

**Table 1:** Statistical outputs from two-way ANOVAs, showing the effects of temperature, the presence of predatory burbot and their interaction term on plasma cortisol levels and telencephalic mRNA levels in juvenile brown trout. Text written in bold denotes statistically significant differences

	Temperature	Predator (burbot)	Temperature × Predator
Cortisol	*F*_1,76_ = 3.11, *P* = 0.08	***F***_**1,76**_ **= 7.22,** ***P*** **= 0.009**	*F*_1,76_ = 0.02, *P* = 0.88
5-HT1Aα	***F***_**1,75**_ **= 13.63,** ***P*** **< 0.001**	*F*_1,75_ = 1.80, *P* = 0.18	*F*_1,75_ = 0.03, *P* = 0.87
5-HT1Aβ	*F*_1,75_ = 3.83, *P* = 0.054	*F*_1,75_ = 2.66, *P* = 0.11	***F***_**1,75**_ **= 4.67,** ***P*** **= 0.034**
AVTR	*F*_1,75_ = 2.67, *P* = 0.11	*F*_1,75_ = 0.10, *P* = 0.75	*F*_1,75_ = 0.81, *P* = 0.37
CALM	*F*_1,75_ = 0.70, *P* = 0.41	*F*_1,75_ = 1.52, *P* = 0.22	*F*_1,75_ = 0.21, *P* = 0.65
CRF1	*F*_1,75_ = 0.01, *P* = 0.92	*F*_1,75_ = 0.25, *P* = 0.62	*F*_1,75_ = 2.14, *P* = 0.15
CRFR1	***F***_**1,75**_ **= 13.22,** ***P*** **= 0.001**	*F*_1,75_ = 0.31, *P* = 0.58	*F*_1,75_ = 1.77, *P* = 0.19
CRHBP	***F***_**1,75**_ **= 12.78,** ***P*** **= 0.001**	*F*_1,75_ = 1.18, *P* = 0.28	*F*_1,75_ = 0.39, *P* = 0.54
EPD1	***F***_**1,75**_ **= 42.20,** ***P*** **< 0.001**	*F*_1,75_ = 0.01 *P* = 0.97	*F*_1,75_ = 0.04, *P* = 0.84
GABARAP	***F***_**1,75**_ **= 83.76,** ***P*** **< 0.001**	*F*_1,75_ = 2.64, *P* = 0.11	*F*_1,75_ = 3.31, *P* = 0.07
GRɑ	***F***_**1,75**_ **= 7.69,** ***P*** **= 0.007**	*F*_1,75_ = 0.89, *P* = 0.35	*F*_1,75_ = 2.27, *P* = 0.14
GRβ	***F***_**1,75**_ **= 9.74,** ***P*** **= 0.003**	*F*_1,75_ = 0.15, *P* = 0.90	*F*_1,75_ = 0.62, *P* = 0.43
GRγ	***F***_**1,75**_ **= 14.50,** ***P*** **< 0.001**	*F*_1,75_ = 0.13, *P* = 0.72	***F***_**1,75**_ **= 4.00,** ***P*** **= 0.049**
GSR	***F***_**1,75**_ **= 14.171,** ***P*** **< 0.001**	*F*_1,75_ = 0.34, *P* = 0.56	*F*_1,75_ = 0.86, *P* = 0.36
MHC1	***F***_**1,75**_ **= 10.6,** ***P*** **= 0.002**	*F*_1,75_ = 0.001, *P* = 0.98	*F*_1,75_ = 1.18, *P* = 0.28
MR	***F***_**1,75**_ **= 34.86,** ***P*** **< 0.001**	*F*_1,75_ = 1.62, *P* = 0.21	*F*_1,75_ = 0.18, *P* = 0.67
RBP1	*F*_1,75_ = 3.21, *P* = 0.08	***F***_**1,75**_ **= 5.69,** ***P*** **= 0.02**	*F*_1,75_ = 0.03, *P* = 0.86

### Telencephalic gene expression

**Fig. 3:**
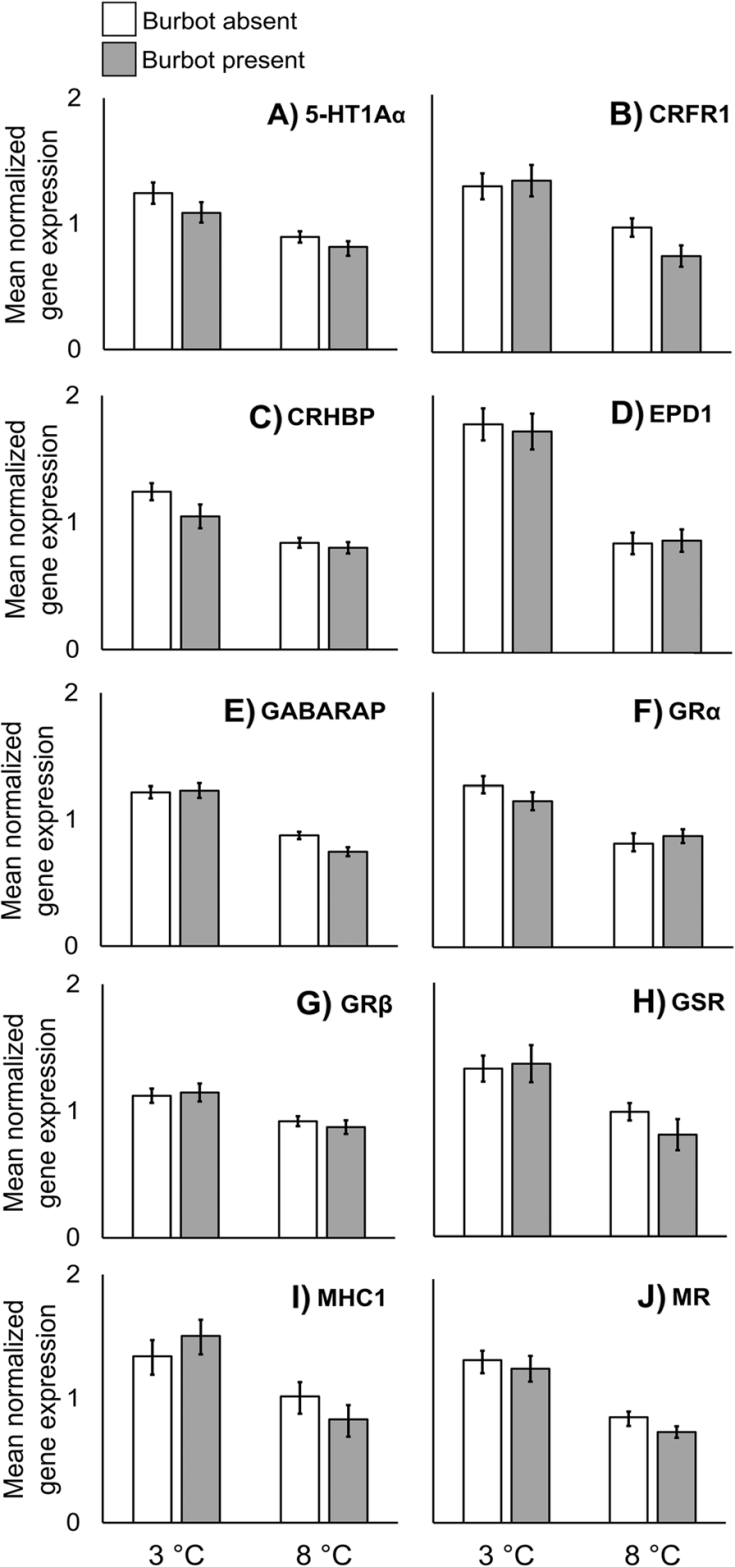
Mean telencephalic gene expressions, measured as relative mRNA levels, of 5-HT1Aα, CRFR1, CRHBP, EPD1, GABARAP, GRα, GRβ, GSR, MHC1 and MR in juvenile brown trout (*Salmo trutta*) (N = 79, see Table 3) at 3 and 8 °C in the presence and absence of predatory burbot (*Lota lota*). Only plots in which there were significant temperature effects (*P* < 0.01) in two-way ANOVAs are shown. Error bars denote ±1 s.e

Temperature had a significant effect on mRNA levels of 5-HT1Aα, CRFR1, CRHBP, EPD1, GABARAP, GRα, GRβ, GSR, MHC1 and MR (Table 1). All these genes exhibited the same expression pattern: trout had higher mRNA levels at 3 than at 8 °C (Fig. 3). There was no significant effect of the predator or of the temperature × predator interaction term for any of these genes (Table 1).

**Fig. 4:**
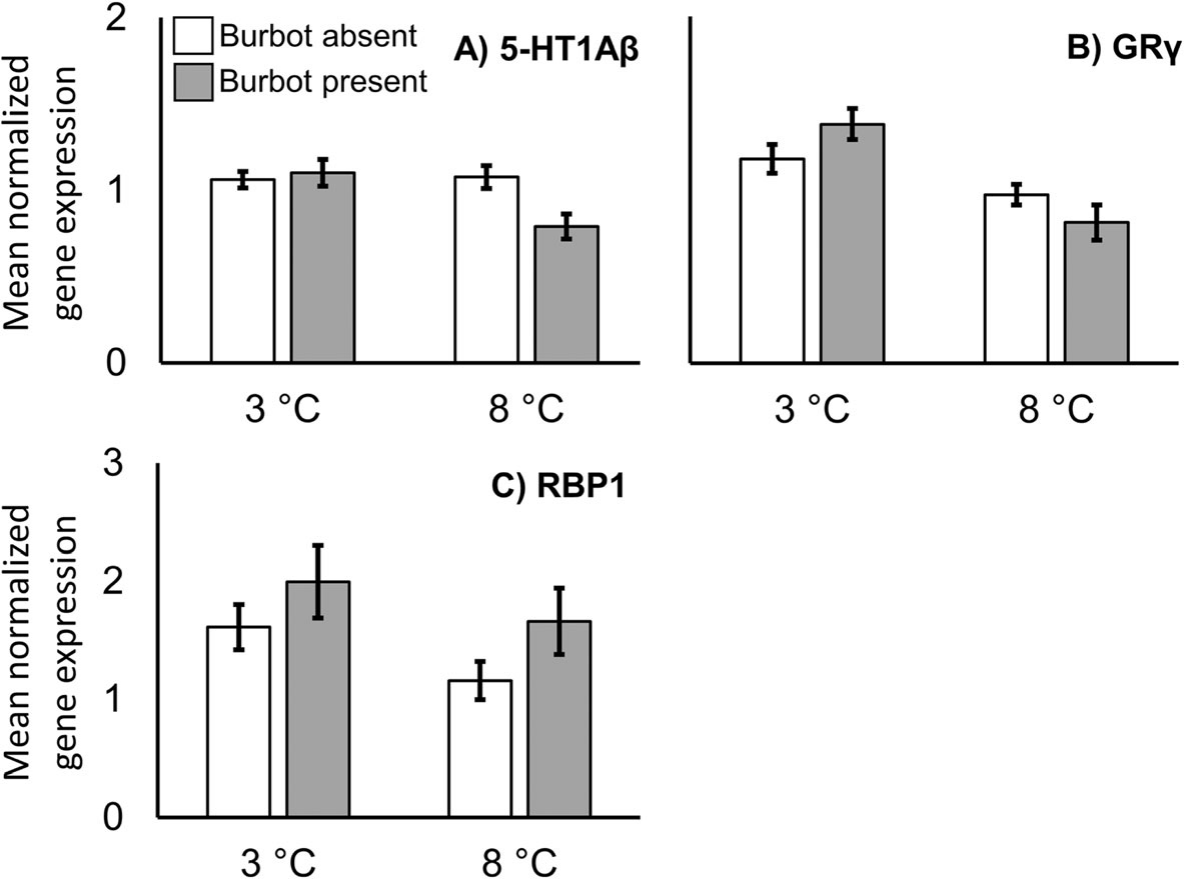
Mean telencephalic gene expressions, measured as relative mRNA levels, of 5-HT1Aβ, GRγ and RBP1 in juvenile brown trout (*Salmo trutta*) (N = 79, see Table 3) at 3 and 8 °C in the presence and absence of predatory burbot (*Lota lota*). Only plots in which there were significant predator or temperature × predator effects (*P* < 0.05) in two-way ANOVAs are shown. Error bars denote ±1 s.e

There was no significant effect of temperature (albeit marginally non-significant, Table 1) or predator presence for 5-HT1Aβ, but there was a statistically significant temperature × predator interaction effect (Table 1). 5-HT1Aβ mRNA levels were significantly lower in the 8 °C/burbot treatment compared to in the other three experimental treatments (Fig. 4a). For the expression of GRγ, we found no effect of the predator, but both significant temperature and temperature × predator interaction effects (Table 1). At 3 °C, the expression of GRγ mRNA was higher in the presence than in the absence of the predator. At 8 °C, however, the expression pattern was reversed (Fig. 4b).

We found a significant positive effect of predator presence on the expression of RBP1 (Table 1, Fig. 4c). Temperature and the temperature × predator interaction term had no significant effect on RBP1 mRNA expression (Table 1). We did not find any significant effects of temperature, predator presence or their interaction term on mRNA levels of AVTR, CALM and CRF1 (Table 1).

## Discussion

Anti-predator behaviours in the presence of piscivorous fish have previously been observed in juvenile brown trout under winter conditions [11, 12]. In the present study, we investigated whether or not anti-predator behaviours are accompanied by changes in plasma cortisol and mRNA levels of stress-related genes. We found elevated plasma cortisol levels in the presence of burbot. In addition, our study demonstrated that for 11 of the analysed 16 genes, trout had higher mRNA levels at 3 than at 8 °C, and that mRNA levels for one gene coding for retinol binding protein 1 was significantly higher in the presence of burbot. Moreover, there were interaction effects between temperature and predator presence for the expressions of one glucocorticoid and one serotonin receptor gene. Hence, we show that anti-predator behaviours appear to be reflected by altered cortisol levels and brain mRNA coding for proteins involved in physiological stress responses.

Considering the observed differences in mRNA levels between temperature treatments, these responses are not likely related to thermal stress, but may simply reflect temperature-induced physiological processes [1, 3]. Gene expression of EPD1, one of the many genes we studied with higher mRNA levels at 3 than at 8 °C, increases when fish experience environmental stress [56, 57]. Moreover, EPD1 activation in common carp (*Cyprinus carpio*) and zebrafish (*Danio rerio*) is strongly associated with cold acclimation, where increased EPD1 mRNA levels at reduced temperatures are stable until the temperature changes [56]. These findings are consistent with our observation of brown trout EPD1 mRNA levels. Hence, our results lend support to the notion that thermal effects on processes related to physiological stress and immune function in poikilotherms can play a major role [56]. Thus, the present study suggests that elevated winter temperatures result in reduced expression of stress-related genes in juvenile brown trout, with possible benefits for the fish if low winter temperatures induce chronic physiological stress [53, 54]. Indirect effects of mild winters should not be omitted however, for instance if high winter temperatures lead to loss of surface ice cover, possibly making stream fishes more susceptible to terrestrial predators.

Even though trout exhibited elevated plasma cortisol levels in the presence of a burbot, mRNA levels of genes that code for CRF and cortisol receptors (CRF1, CRFR1, CRHBP, GRα, GRβ, GRγ and MR) did not differ between treatments with and without the predator. One possible explanation for this result could be that the expressions of these genes normally occur over a different time interval than the one tested in this study. In sockeye salmon (*O. nerka*), increased CRF mRNA expression occurs 8 h after acute stress [70], and in zebrafish, upregulation of MR after an acute stressor occurs after 24 h [71]. Hence, the possibility that mRNA expression and increased cortisol levels do not occur simultaneously could explain why cortisol levels differed significantly between predator treatments in our study, whereas no effects were detected for mRNA levels of CRF or other cortisol-related genes. In contrast, in a study where rainbow trout were subjected to a simulated attack from an avian predator, fish had elevated CRF mRNA levels after the attack, but did not exhibit increased plasma cortisol levels [62]. Studies that consider wider time spans, as well as differences in gene expression depending on what kind of stressor fish experience, may be necessary to clarify these mechanisms.

RBP1 mRNA levels were higher in the presence of burbot than in its absence. RBP1 is involved in the distribution of retinol (vitamin A), which in the form of retinoic acid affects neural plasticity [59]. In zebrafish, physiological stress affects processes related to neural plasticity [72], with subsequent effects on behaviour [30]. High levels of RBP1 mRNA in the presence of a predator may be related to neuroendocrine regulation and altered behaviour. CALM is also associated with neural plasticity and may be affected by stress [59, 61]. In our study however, temperature and predator presence did not influence brain gene expression of CALM. As the functions of both RBP1 and CALM in modulating fish behaviour are not well understood, further studies are needed to elucidate how these processes are involved in anti-predator responses.

Aggression and dominance are often associated with boldness and risk-taking behaviours [73]. In salmonids, GABARAP and EPD1 are involved in both aggression [55, 58] and anti-predator responses [62]. 5-HT and AVT are also related to aggression and dominance in fish [42, 51, 74], and the role of 5-HT in the inhibitory control of aggressive and dominant behaviours seems to be evolutionarily conserved in many animal taxa [75–77]. In our study, 5-HT1Aβ mRNA levels were significantly lower in the presence than in the absence of a burbot at 8 °C, but not at 3 °C. As we were not able to correlate behaviour with gene expressions in this study, however, we cannot infer that the variation in 5-HT1Aβ expression is reflected in trout behaviour. In fact, the results from Filipsson et al. (2019) [12], a study with similar temperature and predator treatments as in this study, did not find temperature-dependent behavioural responses of trout to the presence of burbot.

We used three primer pairs for glucocorticoid receptors, although there is evidence for only two types of glucocorticoid receptors in teleosts (GR1 and GR2) [78, 79]. We did so as none of the previous studies from which we retrieved the primer sequences examined gene expression for brown trout. Instead, they used the closely related Atlantic salmon (*S. salar*) (GRα in the present study) [61] and rainbow trout (GRβ and GRγ in the present study) [40], and since we had no way of knowing the relevant primers, we elected to run all three. All GR-genes showed major differences in mRNA levels between temperature treatments, as did many other genes in this study. The analysis of GRγ also exhibited a significant interaction effect between temperature and predator presence, although with a *P*-value close to alpha (*P* = 0.049). Considering the interaction effects were non-significant for the analyses of GRα and GRβ, we believe that the interaction effect found for GRγ should be interpreted with caution.

## Conclusions

Our study provides new insight into the regulation of plasma cortisol levels and brain gene expression in juvenile brown trout at different temperatures in the presence of a winter-active predatory fish. Our results demonstrate that elevated temperatures can potentially have broad effects on physiological stress responses in fish. We also show that both thermal effects and anti-predator responses are detectable at the gene expression level. Future studies on both long- and short-term temperature effects on behaviour and gene expression, for instance in light of ongoing climate change, are needed to elucidate how stress responses may be affected in an altered climate. Gene expression encompasses more than mRNA levels, as it includes the array of processes that transfer information from gene to functional protein. It may be a fruitful approach to include analyses of gene end products (i.e. proteins) to determine the biological significance of changes in mRNA levels. Hence, studies of relationships between mRNA levels, protein levels and functions and behaviour can be valuable when evaluating potential effects of climate change.

## Methods

### Study fish and husbandry

We retrieved 120 one-summer-old F_1_ hatchery-reared brown trout originating from the River Rottnan, Sweden, from Gammelkroppa hatchery (Gammelkroppa Lax AB) in late January 2018, and transported them to Karlstad University, Sweden. Trout mean wet mass ± 1 s.d. was 8.26 ± 2.3 g, (min - max = 4.33–14.47 g) and mean total length ± 1 s.d. was 100 ± 9 mm, (min - max = 82–121 mm). Upon arrival, trout were acclimated from 1 °C to 3 °C during a 48-h period and evenly distributed into four 200 L aquaria with a water temperature of 3 °C. In two of the aquaria, we kept the temperature constant at 3 °C. In the remaining two, we increased the temperature gradually to 8 °C during a two-week period (+ 0.5 °C every weekday). Trout were allowed to acclimate to aquarium conditions for 3 weeks before the experiment commenced. The photoperiod was set to 10 h daylight (07:00–17:00 h) and 14 h darkness. Water in the aquaria was constantly filtered and aerated (EHEIM 2217 Classic canister filter; Eheim GmbH & Co KG, Deizisau, Germany) and chilled (Teco TK 2000, Teco, Ravenna, Italy), and 25% of the water was changed every week. We fed trout thawed, previously frozen, red chironomids (0.5% of their body mass twice per week).

In the study, we used two burbot (360 and 375 mm) captured in February 2018 at the mouth of the River Klarälven close to Lake Vänern, Sweden (59°21.908′N 13°33.071′E) using fish traps. The burbot were transported to Karlstad University and acclimated from 1 °C to 3 and 8 °C, respectively, over a one-week period in net cages (100 × 50 cm) inside two 7-m-long stream flumes. Burbot were thereafter acclimated to aquarium conditions for 1 week before the experiment commenced. The same photoperiod was used as for the trout. We fed the burbot three thawed, previously frozen, brown trout (individual trout mass ~ 5 g) every week.

### Experimental procedure

We conducted the experiment from mid-February to mid-April 2018. We used a 2 × 2 experimental design, testing the effects of temperature and predation risk: 3 °C with burbot, 3 °C without burbot, 8 °C with burbot, 8 °C without burbot. Trout size did not differ significantly between treatments (one-way ANOVA, *P* = 0.995 and *P* = 0.773 for trout wet mass and total length, respectively). All trials were conducted in eight 130 L aquaria (65 × 50 × 40 cm). Water depth in the aquaria was 20 cm and the bottom substrate consisted of gravel (5–20 mm). We covered all four sides of each aquarium with black sheeting. In addition, a curtain shielded the aquaria from outside disturbance during the experiment. We used four aquaria in each trial, one for each experimental treatment. The aquaria we used for each trial were predetermined from a randomization protocol. In half of the aquaria, the water temperature was 3 °C and in the remaining aquaria 8 °C. Into each of the four aquaria, a transparent plastic chamber (38 × 27 × 27 cm) was inserted for holding the trout. The transparent chambers had 5 mm diameter holes on each end, ensuring that trout could both see and smell the burbot, but not interact physically. Thus, one trout was placed into each of the chambers, and one burbot was placed in two of the aquaria (one at 3 °C and one at 8 °C), outside of the plastic chambers. In preparation for an experimental trial, we randomly selected trout from their respective holding aquaria (3 and 8 °C), and thereafter anaesthetized them with tricaine methanesulfonate (MS-222, Pharmac Ltd., UK, 0.25 g L^− 1^) and measured their total length (mm) and mass (0.01 g). Each trout was placed in an experimental aquarium containing water corresponding to the trout’s acclimation temperature.

At ~ 18:00 h, we placed the trout in the experimental aquaria and left them overnight (with the same light regime as previously described). We subjected trout to the experiment for 20 h, to ensure that the measured physiological stress response not was an artefact of handling the fish or the novel environment to which we introduced trout. Further, burbot is a nocturnal species, so leaving trout with burbot overnight suggests that trout were subjected to the predator under the light conditions when it usually is active [80]. After ~ 20 h, we hand-netted the trout and placed them in a lethal dose (1.5 g L^− 1^) of tricaine methanesulfonate (MS-222, Pharmac Ltd., UK). Trout were euthanized at the same time every day (14:00 ± 1 h) to control for potential diel variation in plasma cortisol and mRNA levels [81]. Trout exhibited loss of equilibrium after < 30 s and ceased opercular ventilation after < 60 s. When no opercular beats could be observed for 10 s and when trout did not respond to strong tactile stimuli, they were euthanized by decapitation and exsanguination. We immediately drew blood samples from the caudal vein by tail ablation, using 75 μl heparinized hematocrit tubes, which were centrifuged for 3 min at 1500 rpm (Hettich D-7200, Hettich GmbH & Co, Tuttlingen, Germany). After centrifugation, blood plasma was isolated and immediately frozen at − 20 °C. Following the blood sampling, we dissected the telencephalon, placed it in 5 ml RLT Plus Buffer (Qiagen, Hilden, Germany) with 2-mercaptoethanol (Sigma-Aldrich, Stockholm, Sweden) and homogenized the tissue using a tissue disruptor with disposable probes (TissueRuptor II, Qiagen, Hilden, Germany). We thereafter extracted RNA using RNeasy plus mini kits (Qiagen, Hilden, Germany), in accordance with the supplier’s manual, eluted in 30 μl water and stored at − 20 °C until use.

### Cortisol measurements

Plasma cortisol concentrations were measured using a competitive enzyme-linked immunosorbent assay (detection range 0–1000 ng ml^− 1^; EKU03476, Cortisol (Cor) ELISA kit, Biomatik, Ontario, Canada), carried out in accordance with the supplier’s manual. We performed a negative control for unspecific degradation of substrate by endogenous plasma peroxidase for each sample.

### Gene expression measurements

We assessed the concentration and quality of RNA in each sample with spectrophotometry (Tecan infinite M200 PRO, Tecan trading AG, Männedorf, Switzerland). We synthesized cDNA using RT^2^ First Strand Kits (Qiagen, Hilden, Germany), adding 500 ng extracted RNA. We diluted the cDNA 1:10 and stored it at − 20 °C. For the gene expression analysis, we selected 16 target genes related to physiological stress responses (including cortisol, serotonin, vasotocin and immune function genes) (Table 2). Primer sequences were obtained from previously published studies [40, 61, 63] and were synthesized by Invitrogen (ThermoFisher Scientific, USA). We performed Quantitative Real-Time PCR (qPCR) with a StepOnePlus Real-Time PCR System (Applied Biosystems, ThermoFisher Scientific, USA), using RT^2^ SYBR Green ROX qPCR Mastermix (Qiagen, Hilden, Germany) in accordance with instructions from the manufacturer’s protocol. The qPCR reactions were performed in a total volume of 12.5 μl with 4 μl cDNA diluted 1:10 and 200 nM of forward and reverse primers. We analysed samples in duplicates and included negative controls. The qPCR protocol consisted of initial denaturation at 95 °C for 10 min, followed by a two-step cycling protocol (95 °C for 15 s + 60 °C for 60 s) for 40 cycles. Melting curve analyses determined the identity and specificity of the PCR products. Cycle threshold was recorded for each sample, and target mRNA levels were normalized, using β-actin and elongation factor 1α (EF1α) as reference genes (Table 2). Fold changes were calculated using the 2^-ΔΔCT^ method [82].

**Table 2:** Genes, accession numbers and primer sequences used for qPCR analysis. Asterisks denote reference genes

Gene	Accession No.	Forward primer	Reverse primer
5-HT1Aα	AGKD01067361	ATGCTGGTCCTCTACGGGCG	CGTGGTTCACCGCGCCGTTT
5-HT1Aβ	DY694524	TTGATCATGCGTTCCCAGCCGA	AAAGGAATGTAGAACGCGCCGA
AVTR	AGKD01053513	ACGGGTTCATCTGCCACAGCA	TGACAGTTCTCAATTTCGCTCTGGA
CALM	BT057678	TGCTGCAGAGCTGCGTCACG	AGCCTCCCGGATCATCTCATCCA
CRF1	NM_001141590	CACACCCACATCCTAGGCTACTCAA	TAGCGGGGTTGGAAGGCACCA
CRFR1	AKD01020666	ATCATCCATTGGAACCTGAT	ATCCAGAAGAAGTTTGTCAC
CRHBP	NM_001173799	TTGAGAAGCGTGCGGTGCGT	AGCTGCTCTCGGAAAGTCCCCT
EPD1	NM_001140909	TCTGTGAGGGTGTGGAGCTGGAG	TTGGTTGGTTGGTTGGGGCTG
GABARAP	NM_001142717	ACTCCCCCTCCTTCCCTCATCCA	ATCCCCATCTCGGCGACCCG
GRα	GQ179974	TGGCCTGTATCCCCCACTGCC	CCGCTGGGCTTGGCTGACG
GRβ	NM_001124730.1	ACGACGATGGAGCCGAAC	ATGGCTTTGAGCAGGGATAG
GRγ	NM_001124482.1	TGGTGGGCTGCTGGATTTCTGC	CTCCCTGTCTCCCTCTGTCA
GSR	BG934480	CCAGTGATGGCTTTTTTGAACTT	CCGGCCCCCACTATGAC
MHC1	AF504021	AATGGATCGCCCCAACGCCA	CTGTCGCGTGGCAGGTCACT
MR	AGKD01011423	AGCTGGCTGGGAAACAGATGA	TCAGGGTGATTTGGTCCTCTATGG
RBP1	NM_001140773.2	GTGGCGGGGCCCTACGCTAT	TCCTGTGCCCAGCATGTCGC
β-actin*	BG933897	CCAAAGCCAACAGGGAGAAG	AGGGACAACACTGCCTGGAT
EF1α*	NM_001124339.1	GCAGGAAAAGAACCCAACG	AGTTACCAGCAGCTTTCTTCC

*5-HT1Aα* serotonin 1a-like receptor; *5-HT1Aβ* serotonin 1b-like receptor; *AVTR* arginine-vasotocin receptor; *CALM* calmodulin; *CRF1* corticotropin-releasing factor 1; *CRFR1* corticotropin-releasing factor receptor 1; *CRHBP* corticotropin-releasing factor binding protein; *EPD1* ependymin; *GABARAP* gamma-aminobutyric acid receptor-associated protein; *GRα* glucocorticoid receptor; *GR*β glucocorticoid receptor 1; *GRγ* glucocorticoid receptor 2; *GSR* glutathione reductase; *MHC1* major histocompatibility complex 1; *MR* mineralocorticoid receptor; *RBP1* retinol binding protein 1; *β-actin* β-actin; *EF1α* elongation factor 1α

### Statistical analyses

Our original design was balanced with equal numbers of replicates of the four experimental treatments. Initially, we used four burbot in this study. Two burbot did however exhibit strong symptoms of stress and deteriorating health during the experiment. To ensure the well-being of these animals, we removed them from the experiment and consequently excluded all cortisol and gene expression data generated from trials with these burbot. Inadequate blood sampling of four trout and low RNA concentrations in four samples (< 100 ng μl^− 1^) also reduced the sample size. Hence, replicates in each experimental treatment varied between 9 and 28 (Table 3).

**Table 3:** The number of juvenile brown trout used to generate the data presented in this study (i.e. final sample size)

	Cortisol		mRNA	
	Burbot present	Burbot absent	Burbot present	Burbot absent
3 °C	15	28	15	27
8 °C	9	28	9	28

We used a multivariate approach to explore the data. A detrended correspondence analysis indicated axis gradient lengths of < 1, suggesting that a linear method was appropriate. We conducted a variance-partitioned redundancy analysis (RDA), using temperature and predator treatments as explanatory factors, to determine how much of the observed variation that could be explained by the experimental treatments. Multivariate analyses were performed in Canoco 5 (Microcomputer Power, Ithaca, NY, USA). To examine effects on cortisol and individual gene expressions, we subsequently analysed the data using two-way ANOVAs. Cortisol and gene expression data were first tested for normality and homogeneity of variances using the Kolmogorov-Smirnov test and Levene’s test for equality of variances. Data that did not meet these assumptions (Cortisol, CALM, 5-HT1Aα, CRF1, CRFR1, GABARAP and MR) were log_10_-transformed. All models used temperature (3 and 8 °C) and predator treatment (burbot presence and absence) as fixed factors, including the interaction term. All univariate analyses were conducted in IBM SPSS Statistics 24 (IBM, Armonk, NY, USA).

## Data Availability

The datasets used and analysed during the current study are available from the corresponding author on request.
